# Antitumor Vaccines Based on Dendritic Cells: From Experiments using Animal Tumor Models to Clinical Trials

**Published:** 2017

**Authors:** O. V. Markov, N. L. Mironova, V. V. Vlassov, M. A. Zenkova

**Affiliations:** Institute of Chemical Biology and Fundamental Medicine SB RAS, Lavrentieva Ave. 8, Novosibirsk, 630090 , Russia

**Keywords:** dendritic cells, antitumor vaccines, delivery of tumor antigens, murine tumor models, clinical trials

## Abstract

The routine methods used to treat oncological diseases have a number of drawbacks, including non-specific action and severe side effects for patients. Furthermore, tumor diseases are associated with a suppression of the immune system that often leads to the inefficiency of standard treatment methods. The development of novel immunotherapeutic approaches having specific antitumor action and that activate the immune system is of crucial importance. Vaccines based on dendritic cells (DCs) loaded with tumor antigens *ex vivo *that can activate antitumor cytotoxic T-cell responses stand out among different antitumor immunotherapeutic approaches. This review is focused on analyzing different methods of DC-based vaccine preparation and current research in antitumor DC-based vaccines using animal tumor models and in clinical trials.

## INTRODUCTION

Dendritic cells (DCs) are professional antigen-presenting cells whose key function is antigen capture, processing, and presentation to naïve T cells to activate an immune response against the captured antigen. The unique ability of DCs to activate CD4+ T helper cells and CD8+ cytotoxic T lymphocytes (CTL) that makes them responsible for the direction of immune responses has attracted increased attention in the development of antitumor vaccines that can exhibit specific activity against certain types of tumors. The discovery of tumor-associated antigens (TAAs), i.e., proteins whose overexpression is specific only to certain tumor types, was an incentive in their application for loading DCs. The TAAs of tumor types such as melanoma (gp100, Melan-A/Mart-1, tyrosinase, MAGE-1 [1, 2]), prostate cancer (PSA [3], PSCA [4]), etc. are known.

Today, antitumor DC-based vaccines are actively studied using both murine models *in vivo *and clinical trials. In 2010, the U.S. Food and Drug Administration approved the first therapeutic vaccine, Sipuleucel-T, against castration-resistant prostate cancer based on DCs loaded with a recombinant fusion protein consisting of prostatic acid phosphatase (PAP) and a granulocyte-macrophage colony-stimulating factor (GM-CSF), which so far remains the only one worldwide [5]. Hence, there are good reasons to develop highly efficient antitumor immunotherapy approaches based on the application of modified dendritic cells in the near future.

This review focuses on strategies using DCs activated by various TAAs both in murine tumor models *in vivo *and in clinical trials.

## PREPARATION OF DC-BASED ANTITUMOR VACCINES

The approaches to antitumor therapy using DCs can be classified into four main groups: (1) injections of DCs loaded with tumor-associated antigens *ex vivo*, (2) systemic administration of tumor-associated antigens to load DCs *in vivo*, (3) injections of non-modified mature DCs, and (4) injections of DC-derived exosomes. In this review, we discuss the conventional DC-based vaccines prepared by loading DCs with tumor-associated antigens *ex vivo*.

Bone marrow-derived cells (for the murine models) and peripheral monocytes (in clinical trials) are commonly used as DC precursors (pre-DCs) when preparing DC-based vaccines. The routine method for the production of DC-based vaccines involves incubation of pre-DCs in the presence of cytokines GM-CSF and IL-4 for 6–8 days, loading immature DCs with tumor-associated antigens, and subsequent activation of dendritic cell maturation using inflammatory cytokines (TNF-α, IL-1β, IL-6, IFN-γ, etc.) or xenogenous factors: LPS (bacterial lipopolysaccharide), OK-432 (low-virulence strain of *Streptococcus pyogenes*), KLH (hemocyanin from mollusk *Fissurella apertura*), etc.

The effectiveness of the antitumor immune response activated by modified DCs is strongly affected by the TAAs used to load immature DCs. Tumor lysates [6-9], synthetic tumor-specific peptides [10-13], tumor proteins [14], apoptotic tumor cells [15], nucleic acids (DNA [16], mRNA [17], total tumor RNA [18, 19]), and viral vectors [15, 20] encoding TAAs as well as immune-stimulating molecules (IL-12 [21, 22]), proliferation factors (GM-CSF [23]), and chemotactic signals (limphotactin [24]) are used as sources of TAAs.

Immature DCs can capture tumor antigens via a number of mechanisms, such as phagocytosis, macropinocytosis, receptor-mediated endocytosis, etc. Hence, tumor-associated antigens of protein nature (proteins, peptides, and lysates) or apoptotic tumor cells are delivered into DCs by passively adding TAAs to immature DCs.

The delivery of nucleic acids (NAs) encoding TAAs requires more complex approaches. NAs are hydrophilic polyanionic molecules that interact with the negatively charged plasma membrane with a poor efficiency and cannot penetrate the cells through the hydrophobic lipid bilayer of the plasma membrane. Furthermore, unprotected NAs are rapidly degraded by nucleases in body fluids. It is also known that free mRNAs can interact with Toll-like receptors (TLR3, TLR7, and TLR8), often resulting in undesired activation of the immune system [25]. Therefore, NAs are delivered into DCs using physical methods (e.g., electroporation [16, 26-28] or sonoporation [29, 30]); viral systems (adenoviruses, adeno-associated viruses, retroviruses, lentiviruses, Vaccinia virus, etc. [31-38]); and nonviral systems (polycationic polymers [31, 39-41] and cationic liposomes [28, 42-45]).

## APPLICATION OF TAA-LOADED DENDRITIC CELLS IN THERAPY OF MALIGNANT NEOPLASMS

*Table 1* and *Table 2* summarize the results of the investigations of DC-based antitumor vaccines in murine models (studies carried out in 2010–2015) and clinical trials (2005–2015). When selecting the studies to be listed, we made allowance for the variety of diseases treated with DC-based vaccines and the TAA sources for loading DCs. We would like to take notice of the great diversity of sources of TAAs for loading DCs used in studies on murine tumor models, from the conventional tumor peptides and lysates to neuraminic acid derivatives and living tumor cells. First of all, antigens of protein nature (tumor cell lysates, proteins, and peptides) were used as the main sources of TAAs for loading DCs in clinical trials. Various routes of vaccine administration (intradermal, intravenous, vaccination into the lymph nodes, etc.) were also employed [46].

## IN VIVO EFFICACY OF DC-BASED VACCINES IN MURINE MODELS

Below, we discuss the results of 15 studies focused on DC-based vaccines in murine models and performed in 2010–2015. Eight of them were devoted to therapeutic DC-based vaccines, where DCs were administered to tumor-bearing mice, four studies focused on preventive DC-based vaccines with DCs administered to animals before tumor grafting, and three studies were devoted to both types of DC-based vaccines. The antitumor potential of DCs was studied in murine tumor models such as colorectal cancer [47, 48], hepatocellular carcinoma [49, 50], Dalton’s lymphoma [51] and EL4 lymphoma [52], FBL3 leukemia[53], 4T1 breast carcinoma [54], B16 melanoma [30, 55-57], Lewis lung carcinoma [58, 59], and SCCVII squamous cell lung cancer models [60] (*Table 1*). In almost all the publications under analysis, DCs were prepared by incubation of bone marrow-derived pre-DCs in the presence of the cytokines GM-CSF and IL-4. Both therapeutic and preventive DC-based vaccines were administered to animals 2 or 3 times with a 7-day interval, preferentially via subcutaneous injections or, less frequently, intraperitoneal or intravenous injections.

**Table 1:** *In vivo* efficacy of DC-based vaccines in animal tumor models

Tumor type	Antigen type	Treatment regimen*	Outcome	Reference
Colorectal cancer	AH1 peptide (gp70 fragment); helper protein ovalbumin (OVA)	SC, 5×10^5^ cells/mouse; twice with a 7-day interval	decreased CT26 tumor size; increased CTL proliferation; increased animal lifespan	[47]
	Adenoviral vectors encoding CEA and SVV; CAR-TAT fusion protein	SC, 1×10^6^ cells/mouse; 2–3 times with a 7-day interval	For DCs simultaneously expressing CEA and SVV: increased in vitro splenocyte reactivity against MC38/CEA2; reduced tumor growth being more efficient in the presence of CAR-TAT	[48]
Hepatocellular carcinoma	Adenoviral vector encoding FAT10; TNF-α	Preventive regimen: SC, 1×10^6^ cells/mouse; 3 times 3 days after subcutaneous injection of Hep3G cells	increased cytotoxic CTL response against Hep3G; decreased Hep3G tumor size; increased animal lifespan	[49]
	Ca9-AbOmpA fusion protein or RENCA-Ca9 cell lysate	SC, 1×10^6^ cells/mouse; 3 times with a 7-day interval	decreased tumor progression 1.5–3-fold increased in vitro secretion of IL-2, IFN-γ, and TNF-α by T cells; increased splenocyte reactivity against RENCA-Ca9	[50]
Dalton’s lymphoma	Dalton’s lymphoma cell lysate; IL-15; combination with cucurbitacin I, IL-15	IP, 1×10^6^ cells/mouse; 6 times with a 4-day interval 10 IP injections (1 mg/kg) of cucurbitacin I with a 1-day interval during 19 days 5 IV injections of IL-15 (8 μg/kg) on days 25–33	The DCs/lysate/IL-15 + cucurbitacin I group: increased survival time of animals (51 days); survival time in the control group was 22 days; complete healing was not achieved. The DCs/lysate/IL-15 + cucurbitacin I + IL-15 group: increased animal survival rate, 70% of animals were alive by day 60, total tumor elimination and healing. Accumulation of CD4+ and CD8+ T cells in metastases	[51]
EL4 lymphoma	EL4 cell lysate; G1-4A polysaccharide from Tinospora cordifola	Preventive regimen: SC, 5×10^5^ cells/mouse; 3 times with a 7-day interval; therapeutic regimen: SC, 5×10^5^ cells/mouse; on tumor progression days 3, 7, and 10	tumor size decreased 2.2- to 3.8-fold for the preventive regimen; tumor size decreased 2.1–2.6-fold for the therapeutic regimen	[52]
FBL3 leukemia, B16F10 melanoma (modified, expressing N-phenylacetyl- D-neuraminic acid)	GM3NPhAc-KLH; combination with ManNPhAc	Preventive regimen: SC, 1×10^6^ cells/mouse; 3 times with a 7-day interval; IP injection of ManNPhAc (50 mg/kg body weight) 7 times every day after tumor was grafted	increased CTL cytotoxicity against modified FBL3 cells; FBL3 tumor size decreased 2.5-fold; increased animal lifespan; the number of B16F10 lung metastases decreased twofold	[53]
4T1 breast carcinoma	Lysate of 4T1 cells and anti-IDO siRNA	IV, 2×10^6^ cells/mouse; 3 times with a 7-day interval	tumor size decreased twofold; reduced apoptosis of CD4+ and CD8+ T cells; increased CTL proliferation; decreased Treg cell count	[54]
B16 melanoma	mRNA encoding β2m-tumor peptide- TLR4 (electroporation)	Preventive regimen: IP, 2.5×10^6^ cells/mouse, 3 times with a 7-day interval Therapeutic regimen: IP, 2.5×10^6^ cells/mouse, 3 times with a 7-day interval	DC maturation. CTL activation. The preventive regimen ensures complete protection against tumor propagation. The therapeutic regimen ensures increased animal lifespan	[55]
	Total protein extracted from melanoma cells (sonoporation)	Preventive regimen: SC, 1×10^6^ cells/mouse; 2 times with a 7-day interval	The number of lung metastases decreased fourfold	[30]
	Living B16 cells; LPS	Preventive regimen: IV, 5×10^6^ cells/mouse; 2 times with a 14-day interval	Complete elimination of B16 tumor; increased CTL count	[56]
	Living or apoptotic B16 cells, gp10025-33 and TRP181-188 peptides, LPS, and IFN-γ	IV, 5×10^6^ cells/mouse; 2 times with a 7-day interval	the number of lung metastases decreased 14.3-fold (DC-living B16 cells); the number of lung metastases decreased 2–2.7-fold (DC peptides and apoptotic B16 cells)	[57]
Lewis lung carcinoma (LLC)	Adenoviral vector encoding human livin α	Preventive regimen: SC, 5×10^5^ cells/mouse; 3 times with a 7-day interval; therapeutic regimen: SC, 5×10^5^ cells/mouse; 3 times with a 4-day interval	increased CTL cytotoxicity; 100% animal survival rate for the preventive regimen; tumor growth decreased twofold and the survival rate of mice increased for the therapeutic regimen	[58]
	LLC cell lysate	IP, 1×10^5^ cells/mouse; 2 times with a 7-day interval	the number of lung metastases decreased 2- to 7.5-fold	[59]
SCCVII squamous cell lung cancer	Apoptotic SCCVII cells	SC, 1×10^6^ cells/mouse; 2 times with a 7-day interval	the number of lung metastases decreased 3.9-fold; the survival rate of mice increased 2.4-fold; CTL cytotoxicity increased	[60]

^*^ – Treatment according to the therapeutic regimen unless otherwise specified.

Protein antigens (first of all, lysate and the total protein of tumor cells) were the most typically used as a source of TAAs to load DCs. The vaccines being used can be subdivided into (1) DC-based vaccines without additional stimuli (B16 melanoma [30] and Lewis lung carcinoma [59]); (2) DC-based vaccines additionally treated with siRNA against immunosuppressive enzyme indolamine 2,3-dioxygenase (4T1 breast carcinoma [54]) or with plant-based immunostimulatory polysaccharide (EL4 lymphoma [52]); and (3) DC-based vaccines combined with injections of cucurbitacin I that selectively inhibits STAT3 in tumor cells (Dalton’s lymphoma [51]). In addition, AH1 tumor peptide (gp70 fragment) in combination with the non-tumor helper peptide (ovalbumin), whose key function was to increase the stability and efficiency of antigen presentation to T cells by DCs, was used as a source of TAAs for the model of colorectal cancer [47]. In the model of hepatocellular carcinoma, a fusion protein (carboanhydrase 9 linked to the membrane protein of *Acinetobacter baumannii*) was used to load DCs [50].

Apoptotic tumor cells, another appreciably common source of TAAs for loading DCs, were used for the model of SCCVII squamous cell lung cancer [60].

DCs were also modified with genetic constructs: namely, adenoviral vectors encoding TAAs (colorectal cancer [48], hepatocellular carcinoma [49], and Lewis lung carcinoma [58]) or mRNA encoding fusion polypeptide β_2_m-tumor peptide-TLR4 containing TAA linked to the components of both MHC I and the Toll-like receptor TLR4 (B16 melanoma [55]).

N-phenylacetyl-*D*-neuraminic acid, a synthetic derivative of neuraminic acid (the models of FBL3 leukemia and B16 melanoma [53]), and living tumor cells (B16 melanoma model [56, 57]) are the novel sources of TAAs used for the activation of DCs.

All the DC-based vaccines under consideration showed significant efficacy and reduced tumor size 1.5- to 3-fold with respect to the control [47-50, 52-54, 58]; injection of DC-based vaccines loaded with tumor lysate in combination with injections of cucurbitacin I resulted in complete disappearance of Dalton’s lymphoma [51]. In addition, injection of preventive DC-based vaccines transfected with mRNA encoding polypeptide β2m-tumor peptide-TLR4 [55] or prepared using living B16 melanoma cells as a source of TAA [56] fully protected animals against the development of B16 melanoma. Antitumor DC-based vaccines significantly reduced the number of metastases in mice [30, 53, 57, 59, 60], considerably increased the lifespan of tumor-bearing animals [47, 49, 51, 53, 55, 58, 60], and induced the development of a strong antitumor response from cytotoxic T lymphocytes [47-50, 53-56, 58, 60].

Hence, the highly promising results for both the therapeutic and preventive application of DC-based vaccines obtained using murine tumor models attest to their high potential and provide grounds for hoping that efficacious antitumor DC-based vaccines will be designed.

## EFFICACY OF DC-BASED VACCINES IN CLINICAL TRIALS

The promising results obtained using murine models *in vivo *encouraged researchers to proceed to the clinical trials of antitumor DC-based vaccines as early as in the 1990s. The safety of antitumor DC-based immunotherapy has been documented in the clinical trials that have been carried out in the past 20 years. DC vaccination is well tolerated [61] and has minor side effects, such as local inflammation reaction at the injection site and in lymph nodes [62, 63]; manifestations similar to influenza symptoms are sometimes observed [63, 64]. Nevertheless, despite its safety and high potential, immunovaccination of cancer patients with DC-based vaccines in most cases has proved less efficacious than in experiments using murine models. There can be various reasons for this, including the fact that in most studies, DC vaccination was used for terminal patients with extremely aggressive tumors that do not respond to conventional therapy and also the fact that human tumors have a stronger immunosuppressive activity. Although there have not been that many impressive clinical results, further development of antitumor DC-based vaccines continues: our understanding of the DC function is being deepened, novel sources of tumor antigens and immunostimulatory agents for loading and activating DC are being tested, and the potential of combining DC-based vaccines with other approaches is being evaluated.

We have made an attempt to assess the variety and clinical efficacy of DC-based vaccines for tumors of different origins. With this aim in mind, we analyzed the results reported in 20 studies performed in 2005–2015; in most of them, DC-based vaccines were in phase I and II clinical trials (*Table 2*). These studies were conducted for cancers of different nosological entities. Various tumor antigens loaded into DCs, treatment regimens, and combination of DC-based vaccines with other therapeutic approaches were employed.

**Table 2:** DC-based antitumor immunotherapy in clinical trials

Tumor type	Phase	Number of patients	Antigen type; maturation stimulus	Treatment regimen*	Outcome	Reference
Pancreatic cancer	I	10	WT-1 peptide. Combination with gemcitabine.	Days 1, 8, 1: IV injection, gemcitabine (1 g/m2). Days 8 and 22: ID injection, 1×10^7^ DCs. Three cycles	DTH+ response (3/10). HLA/WT-1 tetramer-positive test (6/10). Positive IFN-γ ELISPOT (7/10). No clinical response	[65]
Pancreatic and bile duct cancer	I/II	12	MUC1 peptide; TNF-α*, IL-1β*, IL-16*	ID – SC, 1×10^6^ cells, 3 times with a 21-day interval and once 6 months after the last vaccination	perforin and granzyme expression by CD8+ T cells was increased the mean survival rate increased to 26 months (8/12) and more than 7 years (4/12)	[66]
Glioblastoma	I	21	Peptides MAGE1, TRP-2, gp-100, HER-2, IL-13Rα2; TNF-α*	ID, 1×10^7^ cells, 3 times with a 14-day interval	The median survival time is 40.1 months; the mean progression-free survival time is 16.9 months; the mean overall survival time is 38.4 months; 24-month progression-free survival rate is 43.8%; the overall 36-month survival rate is 55.6%	[67]
Colorectal cancer	I	16	mRNA CEA (electroporation) (5/16) or CAP-1, peptide (11/16); cytokine cocktail 1*	ID–IV, 5×10^6^ cells, 3 times with a 7-day interval	CEA-specific T cells (8/11, peptide group; 0/5, RNA group); increased CEA blood level (7/11, peptide group; 2/5, RNA group); mean progression-free survival time is 18 months (the peptide group) and 26 months (the RNA group)	[68]
Hepatocellular carcinoma	I/II	5	Fusion protein (α-fetoprotein, glypican-3, MAGE-3, cytoplasmic transduction); cytokine cocktail 2*	SC, 4×10^7^ cells, 4 times with a 14-day interval and 2 times on weeks 12 and 14 after vaccination was started	Tumor-specific T-cell (5/5); disease stabilization (1/5)	[69]
Liver cancer (stages III and IV)	I	67	Tumor lysate of autologous and allogeneic tumor cells; TNF-α. Combination with cytokine-induced killer cells (CIKs).	DCs: injected into lymph nodes, >10^6^ cells on days 10 and 12. CIKs: IV injection, >1×10^10^ cells on days 12 and 14	Complete response (0/67); partial remission (5/67); disease stabilization (29/67). DC-CIKs suppress HepG2 cell proliferation	[70]
Myeloid leukemia	I	4	Apoptotic leukemia cells; KLH, OK432*	ID, 5 times with a 14-day interval	Antileukemia CD8+ T-cell response (2/4); the leukemia cell count in bone marrow decreased 2.1-fold (1/4)	[71]
T-cell leukemia, lymphoma	I	3	Tax peptides LLFGYPVYV or SFHSLHLLY; TNF-α*, KLH*, OK432*	SC, 5×10^6^ cells, 3 times with a 14-day interval	Tax-specific CTL-response on weeks 16–20 (3/3); complete remission (1/3); partial remission (1/3); disease stabilization (1/3)	[72]
Lymphocytic leukemia	I	15	Autologous apoptotic B cells; TNF-α*	DCs: ID (1×10^7^ cells), 4 times with a 14-day interval, once 14 weeks after the first DC vaccination; GM-CSF: 4 times; after DC vaccination; CP: 2 days prior to DC injection. cohort 1: DCs cohort 2: DCs+GM-CSF cohort 3: DCs+ GM-CSF +CP	Antileukemia CD8+ T-cell response 1) 2/5; 2) 3/5; 3) 5/5	[73]
Osteosarcoma	I	12	Autologous tumor lysate; KLH; PGE2*	ID, 10^5^–10^6^ cells, 3 times with a 7-day interval. After DC-based therapy, SC injections of IL-2 6 times with a 1-day interval	Antitumor CD8+ T-cell response (2/12). No clinical response.	[74]
Ovarian cancer	I/II	11	hTERT 988Y, Her2/ neu 369VV2V9, Her2/ neu 689 and PADRE peptides; Klebsiella pneumoniae*; IFN-γ*	ID, 3.5×10^7^ cells, 4 times with a 21-day interval; IV injection of CP	Disease relapse during vaccination (2/11); Disease relapse after vaccination (3/11); no signs of disease during > 36 months (6/11); the overall 36-month survival rate is 90%	[75]
Melanoma	I	8	Autologous melanoma cell lysate; TNF-α. Combination with tumor-infiltrating T cells. Preliminary chemotherapy.	DCs injected ID, 3 times with a 14-day interval. T cells injected IV, 1–3 times with a 7-day interval 7 days 14 days after last DC vaccination	Complete remission (1/8). Disease stabilization during 2 and 10 months (2/8). Disease progression (7/8)	[76]
	I	30	mRNA encoding fusion protein containing TAAs (MAGE-A1, - A3, -C2, tyrosinase, MelanA/ MART-1 and gp100) and HLA II-targeting sequences (electroporation); Poly (I:C)* or TriMix*. Combination with injections of IFN- α-2b	ID, 2.4×10^7^ cells, 4–6 times with a 14-day interval SC injection of IFN-α-2b 3 times a week	Immune response against melanoma-associated antigens (4/10). Complete remission (10/30). Recurrence of melanoma (20/30). Mean relapse-free survival time is 22 months. The two-year survival rate is 93%. The four-year survival rate is 70%.	[77]
	I	20: 5 – III 15 – IV	Autologous melanoma cell lysate; TNF-α*	SC, 1–5×10^6^ cells, 4 times with a 10-day interval	Increased IFN-γ secretion (10/20); Increased CTL cytotoxicity (4/20); DTH+ response (11/20); Time of tumor progression increased 4.1-fold (the DTH+ response group vs the DTH- response group); survival rate increased 2.9-fold (the DTH+ response group vs the DTHresponse group).	[78]
	II	24	Peptides gp100, tyrosinase, MAGE-A2, MAGE-A3, MART-1, MAGE-A1; KLH*	SC, 1–5×10^7^ cells, 4 times with a 7-day interval, then once after 14 days and 5 times with a onemonth interval.	increased count of tumor-specific CD8+ T cells (18/24); activation of Th1 response (12/24); DTH+ response to TAAs in 41% of patients; DTH+ response to KLH in 64% of patients; patient survival rate increased 1.9-fold; partial response (1/24); disease stabilization (7/24); disease progression (16/24)	[79]
		33	Lysates of cells of allogeneic melanoma lines M44, COLO829, SK-MEL28; IFN-γ*	Near the lymph nodes, 2.5×10^7^ cells, 6 times with a 14-day interval, then twice with a 42-day interval	The count of tumor-specific CD8+ T cells in blood increased (26/33). Complete remission (1/33), partial response (2/33), disease stabilization (6/33)	[80]
Non-small cell lung cancer	III	103	DCs were not loaded with TAAs. Vaccine consisted of DCs and T cells derived from pulmonary lymph nodes and incubated in the presence of IL-2 with peripheral blood T cells added	Group A: 4-month chemotherapy course, DC-based vaccine 1 week after each chemotherapy course + once a month (during 6 months) + once every 2 months (during 14 months). Group B: 4-month chemotherapy course	The overall two-year survival rate in groups A and B is 93.4 and 66.0%, respectively; the overall five-year survival rate in groups A and B is 81.4 and 48.3%, respectively; the relapse-free 2- and 5-year survival rate is 68.5 and 41.4% (group A); 56.8 and 26.2% (group B).	[81]
Prostate cancer	I/II	25	UV-treated LNCaP cells; poly (I : C)*. Combination with chemotherapy.	1. Cyclophosphamide, 7 days 2. DCs, SC injection, 10^7^ cells, 12 times within the first year (2 times with a 2-week interval) 3. Docetaxel every 3 weeks until toxicity is achieved. 4. SC injection of DCs, 10^7 ^ cells, 10 times with a 6-week interval	PSA level decreased by ≥ 50% (8/23) PSA level decreased by 20–50% (5/23) Blood level of Tregs decreased Induction of PSA-specific CTLs. The mean survival time is 19 months.	[82]
	III	127	PAP-GM-CSF, fusion protein	IV, 3.7×10^9^ cells, 3 times with a 14-day interval	Disease progression (115/127); time of disease progression increased 1.2-fold; the mean survival rate increased 1.2-fold	[83]
	III	512	PAP-GM-CSF, fusion protein	IV, 3.7×10^9^ cells, 3 times with a 14-day interval	the mortality risk decreased by 22%; the mean survival rate increased 1.2-fold; the 36-month survival rate increased 1.4-fold; Activation of Th1- response.	[5]

Note: * – factors for dendritic cell maturation; SC – subcutaneous; IP – intraperitoneal; IV – intravenous; ID – intradermal; DTH response – delayed type (type IV) hypersensitivity reaction; KLH – keyhole limpet hemocyanin from Fissurella apertura, OK432 – a mixture of low-virulence group A Streptococcus pyogenes; cytokine cocktail 1 – PGE2, TNF-α, IL-1β, IL-6; cytokine cocktail 2 – PGE2, TNF-α, IL-1β, IL-6, IFN-γ, OK432, poly (I : C); TriMix – mRNA encoding CD40L, CD70 and the constitutively active TLR4; CP – cyclophosphamide; disease stabilization – there are no visible changes in tumor size; disease progression – there is a 20% increase in tumor size; partial response – a 30% decrease in tumor size; and complete response – tumor disappearance.

Three DC-based vaccines have passed phase III trials; one of these vaccines, Sipuleucel-T, used against castration-resistant prostate cancer was later approved by the FDA under the brand name Provenge® [5].

The antitumor potential of DC-based vaccines was evaluated in patients with a cancer of the gastrointestinal tract (liver, pancreatic, and colorectal cancer), brain (glioblastoma), blood (myeloid leukemia, lymphocytic leukemia, lymphoma), bone tissue (osteosarcoma), the reproductive system (ovarian or prostate cancer), skin (melanoma), and lungs (non-small cell cancer) both after the tumors had been surgically resected and patients had undergone conventional chemo- or radiotherapy and in treatment-naïve patients (*Table 2*). The adequacy of DC-based vaccines was evaluated using two criteria: the immunological criterion and the clinical one. The key immunological variables measured in clinical trials of DC-based vaccines were as follows: the immune response against TAAs (type IV hypersensitivity response to tumor antigens (DTH response)), the presence of HLA complexes with tumor antigens on the surface of DCs, the expression of perforin/granzyme by CD8+ T cells, the activity of cytotoxic CD8+ T cells against tumor cells, the level of IFN-γ synthesis by T cells, the regulatory T-cell count in blood and tumor cell count in the bone marrow, the concentration of tumor markers (PSA, CEA) in blood serum, etc. (*Table 2*). The clinical response to immunotherapy with DC-based vaccines was assessed according to the patient survival rate, disease remission/relapse (a 20% increase in tumor size was considered to be a sign of disease progression; no visible changes in tumor size were a sign of stable condition), partial response or partial remission (tumor size reduction by 30%), and complete response or complete remission (tumor disappearance) (*Table 2*).

In most of the analyzed studies, the DCs were derived from peripheral blood mononuclear cells cultured in the presence of cytokines GM-CSF and IL-4. In one case, GM-CSF and IL-13 were used to prepare the DC-based vaccine [79]. Unconventional DC-based vaccines were used as antitumor vaccines undergoing phase III clinical trials: DCs isolated from pulmonary lymph nodes were used in non-small cell lung cancer [81]; the Sipuleucel-T vaccine (a cellular agent isolated from leukapheresis-derived products that included DCs) was used in patients with prostate cancer [5, 83].

Protein antigens (peptides, synthetic proteins, and tumor cell lysates) were used most frequently (15 out of 20 publications) as a source of TAAs to load DCs. Peptide TAAs were used both as a single antigen (WT-1 or MUC1 in the case of pancreatic cancer [65, 66]) and as an antigen mixture (MAGE1, TRP-2, gp100, HER-2, IL-13Rα2 in glioblastomas [67]; gp100, tyrosinase, MAGE-A1, -A2, -A3, MART-1 in melanoma [79]; HTLV-1 Tax peptides in T-cell leukemia and lymphoma [72], and hTERT, Her2/neu, and PADRE fragments, in ovarian cancer [75]). mRNAs encoding a single antigen (CEA in the case of colorectal cancer [68]) or a combination of antigens (MAGE-A1,-A3,-C2, tyrosinase, MelanA/MART-1, and gp100 for melanoma [77]) were also used to load DCs. In the case of hepatocellular carcinoma, DCs were loaded with fusion proteins containing TAAs such as α-fetoprotein, glypican-3 and MAGE-3, each of those connected to a cytoplasmic transduction peptide [69]. Another fusion protein containing PSA coupled to GM-CSF was used for loading the Sipuleucel-T vaccine [5, 83]. Tumor cell lysates were used to prepare DC-based vaccines against osteosarcoma [74] and melanoma [76, 78, 80]; the lysate was most often prepared from autologous tumor cells. DCs were also often loaded with apoptotic tumor cells (e.g., in DC-based vaccines against myeloid [71] and lymphocytic leukemia [73] and prostate cancer [82]). In the DC-based vaccine against non-small cell lung cancer, DCs were not loaded with TAAs at all but were used together with T cells after coincubation in the presence of IL-2 [81].

It is known that injection of immature DCs into a tumor-bearing organism can cause the development of tolerance of the immune system to tumor antigens, ultimately resulting in an even greater tumor progression [84]. Therefore, much attention is paid to agents that stimulate DC maturation in almost all clinical trials of DC-based vaccines. Both single pro-inflammatory cytokines (TNF-α or IFN-γ) and cocktails containing a combination of pro-inflammatory cytokines, prostaglandin E2, and in some cases poly(I:C)oligonucleotides, low-virulence *S. pyogenes *(OK432), bacteria *Klebsiella pneumoniae *or hemocyanin from *F. apertura *(KLH) were used for this purpose (*Table 2*).

Despite the variety of protocols for tumor immunotherapy with DC-based vaccines, common features can also be listed. DCs are preferentially administered either intradermally or subcutaneously 3–4 times with a 7- to 14-day interval. The mean dose is 10^6^–10^7^ DCs. In some cases, DC vaccination can be combined with chemotherapy (gemcitabine for pancreatic tumor [65], cyclophosphamide for lymphocytic leukemia [73] and ovarian cancer [75], and docetaxel for prostate cancer [82]), with the application of other immune cells (e.g., cytokine-induced killer cells; i.e., T cells and natural killers activated by IL-1, IL-2, IFN-γ and anti-CD3 antibodies for hepatic tumors [70]); tumor-infiltrating lymphocytes for melanoma [76]), as well as with injections of cytokines (GM-CSF for lymphocytic leukemia [73], IL-2 for osteosarcoma [74], and IFN-α-2b for melanoma [77]).

It was demonstrated in almost all the studies under consideration that administration of DC-based vaccines activates an antitumor immune response: tumor-specific cytotoxic CD8^+^ T cells are activated; perforin and granzyme expression and IFN-γ production are enhanced; some patients develop a hypersensitivity response to tumor antigens (the DTH response); the regulatory T-cell count decreases, etc. However, despite the substantial immune response, the clinical efficacy of antitumor DC-based therapy is less impressive. The clinical response is either rather weak or absent, which manifests itself in a large number of relapses and tumor progression. Even Sipuleucel-T, the only FDA-approved antitumor DC-based vaccine, exhibits low efficacy. Immunotherapy using this vaccine resulted in remission in none of the patients; disease progression was observed in most cases, although patient survival increased 1.2-fold compared to the placebo group [83]. Hence, a conclusion can be drawn that activation of a tumor-specific immune response after DC vaccination does not necessarily provide significant clinical outcomes. First of all, this can be attributed to the negative effect of the tumor on the immune system. Even provided that antitumor T cells are properly activated by DC-based vaccines, immunotherapy may fail, since the tumor can evade immune surveillance by suppressing the functional activity of immunocompetent cells, including T cells and DCs, via various mechanisms [85].

Tumor remission in a number of patients is indicative of the clinical significance of DC vaccination. Hence, immunotherapy with DCs loaded with a mixture of tumor-specific peptides (hTERT 988Y, Her2/ neu 369VV2V9, Her2/neu 689, and PADRE) in combination with cyclophosphamide injections resulted in the absence of disease symptoms during 36 months in more than 50% of patients with ovarian cancer (6/11); the 36-month survival rate was 90% [75]. This is one of the highest indices of clinical efficacy of DC-based vaccines in the studies covered in this review.

Many remissions were observed in patients with melanoma after they had received DC-based vaccines loaded with a mixture of mRNAs encoding TAAs MAGE-A1,-A3,-C2, tyrosinase, MelanA/MART-1 and gp100 fused with HLA II-targeting sequences, in combination with IFN-α-2b injections [77]. During the mean follow-up (6.4 years), 10 out of 30 patients showed complete remission. The mean relapse-free survival time was 22 months. The mean two- and four-year survival rate was 93 and 70%, respectively. Four out of 10 patients showed an immune response against melanoma-associated antigens [77].

Either complete or partial remission of melanoma was also observed after patients had undergone immunotherapy with DC-based vaccines with lysates of autologous melanoma cells (1 out of 8 patients) [76] or the allogeneic cell lines M44, COLO829, and SK-MEL28 (complete response in one out of 33 patients; partial response, in 2 out of 33 patients) [80]. It is noteworthy that melanoma is used appreciably often in clinical studies of the antitumor activity of DCs and is relatively more susceptible to immunotherapy than other tumor types.

A high efficacy of DC-based vaccines was also observed in a pilot clinical study of DCs against T-cell leukemia and lymphoma with three patients enrolled [72]. DCs loaded with Tax peptides of human T-lymphotropic virus 1 (LLFGYPVYV and SFHSLHLLY) that matured under standard stimulus (TNF-α in combination with the xenogeneic factors KLH and OK432) have been used as DC-based vaccines. After DC vaccination, all three patients showed a significant clinical response: complete remission (1/3), partial remission (1/3), and disease stabilization (1/3). The efficacious clinical response was related to the development of a Tax-specific CTL response in all patients [72].

The survival rate of patients is also an important indicator of the efficacy of antitumor DC vaccination. Almost all clinical trials demonstrate that administration of DC-based vaccines to patients with tumors of different types increases their survival rate and life expectancy compared to patients not treated with a DC-based vaccine. Hence, the most significant increase in the survival rate in the analyzed studies was achieved in patients with pancreatic and bile tract cancers who received DCs that were loaded with MUC-1 peptide and stimulated with the cytokines TNF-α, IL-1β, and IL-16: the mean survival time was more than 7 years in 4 out of 12 patients [66].

Let us discuss three phase III clinical trials of antitumor DC-based vaccines in more detail. In the first study, a vaccine based on DCs and activated killer T cells, in combination with chemotherapy, was used in patients with non-small cell lung cancer [81] after the tumor had been surgically resected. One hundred and three patients were enrolled and divided into two groups: group A received immunochemotherapy, while group B received chemotherapy only. The vaccine was based on DCs and activated killer T cells which were isolated from the contents of lymph nodes residing at tumor sites and cultured in the presence of IL-2; peripheral blood T cells were subsequently added. The overall two-year survival rates in groups A and B were 93.4 and 66.0%, respectively; the overall five-year survival rates were 81.4 and 48.3%, respectively. The two- and five-year relapse-free survival rates were 68.5 and 41.4%; 56.8 and 26.2% in groups A and B, respectively [81].

The Sipuleucel-T DC-based vaccine, which was used for patients with castration-resistant prostate cancer, was assessed in two other trials. However, the results of clinical trials of Sipuleucel-T were less impressive compared to those for other antitumor DC-based vaccines [83]. Sipuleucel-T is a cellular agent isolated from leukapheresis-derived products that included DCs. The cells were loaded with a fusion protein consisting of full-length PAP and full-length human GM-CSF (PAP-GM-CSF). Patients with asymptomatic metastatic hormone-refractory prostate cancer were enrolled. Administration of this vaccine caused disease progression in most patients. Nevertheless, Sipuleucel-T increased the mean survival time 1.2-fold (25.8 months vs 21.7 months in the placebo group) and resulted in the development of immune response to PAP and T-cell response [5, 83]. Soon after these results were published, Sipuleucel-T was approved by the U.S. Food and Drug Administration (FDA USA) for treating patients and commercialized under the trademark Provenge® [86].

## EFFICACY OF ANTITUMOR DENDRITIC CELLBASED VACCINES: QUESTIONS AND ANSWERS

It is clear from the studies discussed above that most of the antitumor DC-based vaccines that have successfully passed clinical trials have limited efficacy. Some researchers believe that the low efficacy of DC-based vaccines can be related to the fact that their effect on patient survival becomes noticeable only some time after treatment [5]. However, in our opinion, the key reason for the low efficacy of DC-based vaccines is the strong immunosuppressing action of the tumor that is ensured by a number of mechanisms. For example, the tumor and the surrounding tissue can reduce the penetration of T cells into the tumor site, reduce granzyme B activity, suppress death receptor CD95 expression by T cells, and induce anergy of activated T cells by enhancing the expression of the inhibitory receptors CTLA-4 and PD-1 (the so-called immune checkpoints) on the T-cell surface [87]. The immunosuppressive activity of CTLA-4 consists in the fact that it is in competition with the standard participant of the immunological synapse, CD28, for binding to the DC-derived costimulatory molecules CD80 (B7.1) and CD86 (B7.2) and the transmission of the inhibitory signal to T cells, thus attenuating the TCR/CD28 signaling pathway of T cells, reducing IL-2 production by T cells, and eventually resulting in cell cycle delay [87, 88]. The PD-1 receptor interacts with the B7-H1 molecules expressed on the tumor cell surface, which also disrupts the TCR/ CD28 signaling pathway, induces the synthesis of anti-inflammatory cytokine IL-10, and eventually results in the activation of immunosuppressive Tregs and apoptosis of tumor-specific T cells [87, 89].

It is reasonable to employ additional methods aimed at reducing the inhibitory effect of the tumor to enhance the efficacy of DC-based vaccines. Thus, CTLA- 4, PD-1, and B7-H1 blocking antibodies combined with antitumor DC-based vaccines will make it possible to reduce the immunosuppressive activity of the tumor, which may significantly increase the antitumor activity of DC-based vaccines. The immune checkpoint inhibitors known today are Ipilimumab [90] for CTLA-4, Nivolumab [91] and Pembrolizumab [92] for PD-1. The FDA has recently approved these monoclonal antibodies for the immunotherapy of metastatic melanoma [93, 94]. B7-H1 blocking antibodies are currently under clinical trials but have not been approved for clinical use yet [95]. Only one study reporting the use of antitumor DC-based vaccines in combination with immune checkpoint inhibitors has so far been published. In this phase II clinical trial, patients with melanoma received a combination of Ipilimumab and DC-based vaccines loaded with TriMix RNA and mRNA encoding melanoma-associated antigens. Very promising results have been obtained: after the therapy course, eight of the 39 patients showed complete remission and seven patients showed a partial response [96]. Clinical trials of antitumor DC-based vaccines in combination with immune checkpoint inhibitors will undoubtedly be forthcoming.

## CONCLUSIONS

Despite the great variety of the mechanisms used by a tumor to evade the immune response, promising results have been obtained for cancer immunotherapy using modified DCs. Experiments using murine models have shown a reduced tumor growth rate, a decline in the number of metastases, an increase in the survival rate of tumor-bearing animals, and initiation of a tumor-specific CTL response [50, 54, 57, 97, 98]. The results of clinical trials of antitumor DC-based vaccines were also fairly good, although less encouraging compared to those obtained using *in vivo *murine models. A plausible reason is that clinical trials in most cases are performed on terminally ill patients when no other therapy shows any effect. Furthermore, the low efficacy of DC-based vaccines can be related to the fact that the human immune system is suppressed by the tumor to a greater extent.

The problems related to the search for the most immunogenic source of TAAs, the insufficient specificity and efficiency of TAA delivery into DCs remain to be solved. This may affect the presentation of processed TAAs bound to complexes with MHC I/II molecules on the DC surface and the weak polarization of antitumor immune responses. Therefore, further development of antitumor DC-based vaccines that would be capable of countering the negative effect of the tumor and its surroundings and initiate an efficient antitumor immune response remains a top priority.

## Abbreviations

Ag: antigen
APC: antigen-presenting cells
GM-CSF: granulocyte-macrophage colony stimulating factor
DCs: dendritic cells
IL: interleukin
IFN: interferon
NA: nucleic acid
TAAs: tumor-associated antigens
PAP: prostatic acid phosphatase
Treg: regulatory T cells
UV: ultraviolet
TNF: tumor necrosis factor
CIK: cytokine-induced killer cells
CTL: cytotoxic T lymphocytes
CP: cyclophosphamide
DTH: delayed type (IV) hypersensitivity
HLA: human leukocyte antigen
KLH: keyhole limpet hemocyanin of F. apertura
OVA: ovalbumin
SVV: survivin
TLR: toll-like receptor
